# Trends in HIV Testing among Adults in Georgia: Analysis of the 2011–2015 BRFSS Data

**DOI:** 10.3390/ijerph13111126

**Published:** 2016-11-11

**Authors:** Benjamin E. Ansa, Sashia White, Yunmi Chung, Selina A. Smith

**Affiliations:** 1 Institute of Public & Preventive Health, Augusta University, CJ-2300 1120 15th Street, Augusta, GA 30912, USA; ychung@augusta.edu (Y.C.); sesmith@augusta.edu (S.A.S.); 2 Medical College of Georgia, Augusta University, 1120 15th Street, Augusta, GA 30912, USA; saswhite@augusta.edu; 3 Department of Family Medicine, Medical College of Georgia, Augusta University, Augusta, GA 30912, USA

**Keywords:** HIV/AIDS, testing, trends, Behavioral Risk Factor Surveillance System, socio-demographic, Georgia

## Abstract

Georgia is ranked fifth highest among states for rates of human immunodeficiency virus (HIV) diagnosis. About 4% of persons living with HIV infection in the United States reside in Georgia, and almost 19% of these people do not know their HIV status. The present study examined the trends and associated factors of HIV testing among adults in Georgia between 2011 and 2015 by analyzing data of the Behavioral Risk Factor Surveillance System (BRFSS). A total of 31,094 persons aged ≥18 years were identified who responded to the question “Have you ever been tested for HIV?” Overall, there were 11,286 (44.2%) respondents who had been tested for HIV, compared to 19,808 (55.8%) who had not. There was a slight decrease in the percentage of respondents who have ever tested for HIV, from 45.6% in 2011 to 43.7% in 2015 (APC (annual percent change) = −0.98, not significant). Factors associated with HIV testing were being female (*p* = 0.004), black (*p* < 0.001), younger than 55 years (*p* < 0.001), single (*p* < 0.001), attaining education level above high school (*p* < 0.001), and earning annual income of $50,000 or less (*p* = 0.028). Overall in Georgia, there has been a slight decline in the temporal trend of HIV testing, and more than half of adults have never been tested for HIV. For reducing HIV transmission in Georgia, enhancing access and utilization of HIV testing should be a public health priority.

## 1. Introduction

Human immunodeficiency virus (HIV) testing contributes to the prevention and control of HIV/AIDS. Following HIV diagnosis, risk behaviors tend to decrease [1,2,3]; people diagnosed with HIV can make decisions that potentially lower HIV transmission risk by avoiding risk behaviors such as unprotected sex and needle sharing [4]. Also, people who test negative for HIV can make decisions to protect themselves from HIV by engaging in safer sex behaviors and in some cases, taking pre-exposure prophylaxis [4]. Enabling individuals to become diagnosed early is a public health priority [5], as late diagnosis of HIV infection can lead to increased morbidity, mortality, and healthcare costs [5].

According to the Centers for Disease Control and Prevention (CDC), an estimated 1.2 million people in the United States (U.S.) are living with HIV, and 13% (156,300) of these do not know they are infected [6]. Each year, nearly 45,000 people are diagnosed with HIV, with 30% of new HIV infections being transmitted by people who are living with undiagnosed HIV [4]. Geographically, the burden of HIV is not evenly distributed. In 2014, the rates (per 100,000 people) of persons diagnosed with HIV infection were the highest in the South (18.5), followed by the Northeast (14.2), the West (11.2), and the Midwest (8.2) [6].

Georgia (GA) with a population of 10,214,860 in 2015, is the second most populous state in the southeast of the U.S. [7]. The population is made up of 62% whites, 32% blacks/African Americans, 9.0% Hispanics/Latinos, and 5.0% other races/ethnicities [7]. With a rate of 27 per 100,000, GA is ranked fifth highest among states in regard to rates of HIV diagnosis. According to the CDC, 1 in 51 Georgians will be diagnosed with HIV in their lifetime (compared to 1 in 670 residents of North Dakota) [8]. Despite comprising only 3.1% of the U.S. population in 2014, 6.4% (2640) of new HIV diagnoses and 4.4% (53,230) of persons living with HIV infection in the U.S. were recorded in GA [9]. About 19% of those living with HIV are unaware of their HIV status, and almost 23% of persons in the state of GA were diagnosed with AIDS within three months, as a result of late testing for HIV [9,10]. This means that they harbored the virus for a long period of time without receiving appropriate treatment that would have prevented further deterioration of their immune system.

In 2006, the CDC recommended that, as part of routine health care, everyone between the ages of 13 and 64 be tested for HIV at least once, with yearly HIV testing for high-risk individuals, in line with the National HIV/AIDS Strategy goal of increasing by 2015 the percentage (to 90%) of persons living with HIV who know their serostatus [11,12]. The purpose of the present investigation was to evaluate the progress in HIV testing in GA by examining the temporal trends and factors associated with testing for HIV among adults residing in GA between 2011 and 2015.

## 2. Materials and Methods

### 2.1. Study Design

This cross-sectional study was done by analyzing nationally representative datasets.

### 2.2. Data Source, Study Participants, and Sampling

The Behavioral Risk Factor Surveillance System (BRFSS) is a nationally representative cross-sectional survey that collects data on U.S. residents in all 50 states, the District of Columbia, and three U.S. territories, regarding their health-related risk behaviors, chronic health conditions, and use of preventive services [13]. GA has been part of the system since it was established in 1984 [14]. Surveys are conducted through phone interviews (landline and cellphone) and more than 400,000 adult interviews are conducted each year, making it the largest continuously conducted health survey system in the world and a useful tool for addressing and developing health promotion activities [13].

Although conducted in different time periods, the surveys used identical methods for recruitment. GA is among the participating BRFSS states that utilize disproportionate stratified sample (DSS) design for their landline samples [15]. Telephone numbers are divided into two groups, or strata, which are sampled separately. The high-density and medium-density strata contain telephone numbers that are expected to belong mostly to households. Whether a telephone number goes into the high-density or medium-density stratum is determined by the number of listed household numbers in its hundred block, or set of 100 telephone numbers with the same area code, prefix, and first two digits of the suffix and all possible combinations of the last two digits. BRFSS puts numbers from hundred blocks with one or more listed household numbers (1 + blocks, or banks) in either the high-density stratum (listed 1 + blocks) or medium-density stratum (unlisted 1 + blocks). The BRFSS samples the two strata to obtain a probability sample of all households with telephones. Cellular telephone sampling frames are commercially available and the system can call random samples of cellular telephone numbers, but doing so requires specific protocols [15]. The basis of the 2011–2015 BRFSS sampling frame is the Telecordia database of telephone exchanges (e.g., 617-492-0000 to 617-492-9999) and 1000 banks (e.g., 617-492-0000 to 617-492-0999). The vendor uses dedicated cellular 1000 banks, sorted on the basis of area code and exchange within a state. The BRFSS forms an interval (K) by dividing the population count of telephone numbers in the frame (N) by the desired sample size (n). The BRFSS divides the frame of telephone numbers into n intervals of size K telephone numbers. From each interval, the BRFSS draws one 10-digit telephone number at random. In the sample design, each state begins with a single stratum. To provide adequate sample sizes for smaller geographically defined populations of interest, however, many states sample disproportionately from strata that correspond to substate regions.

Response rates for BRFSS were calculated using standards set by the American Association of Public Opinion Research (AAPOR) Response Rate Formula 4 [16]. The median survey response rate (%) for all states and Washington, DC, in 2011 was 49.7, and ranged from 33.8 to 64.1 [17]; in 2012 was 45.2, and ranged from 27.7 to 60.4 [18]; in 2013 was 46.4, and ranged from 29.0 to 60.3 [19]; in 2014 was 47.0, and ranged from 25.1 to 60.1 [20]; and in 2015 was 47.2, and ranged from 33.9 to 61.1 [21]. Response rates (%) for GA included in this analysis had a weighted AAPOR response rate of 49.9 in 2011 [17], 53.5 in 2012 [18], 46.5 in 2013 [19], 48.8 in 2014 [20], and 47.6 in 2015 [21].

Secondary analyses of the BRFSS 2011–2015 data were performed to identify persons in GA aged ≥18 years who reported having ever been tested for HIV.

### 2.3. Measures

Respondents were categorized under socio-demographic variables of gender (male or female); age in years (18–24, 25–34, 35–44, 45–54, 55–64, or 65+); race (non-Hispanic (NH) white, NH black, Hispanic, NH other, or NH multiracial); education (<high school, high school/General Educational Development (GED), some post high school, or college graduate); annual income in United States Dollar (USD (<$15,000, $15,000–<$25,000, $25,000–<$35,000, $35,000–<$50,000, or $50,000+)); marital status (married, divorced, widowed, separated, never married, or a member of an unmarried couple); healthcare coverage (yes/no); and HIV high-risk situations (yes/no). HIV high-risk situations included engaging in any of the following behaviors for the past year: use of intravenous drugs, treatment for sexually transmitted disease, giving or receiving money or drugs for sex, or having anal sex without a condom. The type of health coverage was only assessed for the year 2014 these data were available. The outcome variable was participants’ response to the question “Have you ever been tested for HIV?” (yes/no).

### 2.4. Statistical Analyses

Descriptive statistics of socio-demographic variables and HIV high-risk situations related to HIV testing were generated for each year, using frequencies and proportions. Data were weighted using the iterative proportional fitting weighting method (i.e., raking) to adjust for noncoverage, nonresponse, and for differences between sample and population characteristics [22]. Weighted percentages of respondents who had ever been tested for HIV were calculated for each variable category for each year. Joinpoint Trend Analysis software [23,24] was used to calculate the annual percent change (APC) over time. The model is linear on the log of the response for calculating annual percentage rate change. An APC is computed for each of those trends by means of generalized linear models assuming a Poisson distribution. The tests of significance use a Monte Carlo Permutation method. Significant changes include changes in direction or in the rate of increase or decrease. Logistic regression analyses were conducted to examine the association between socio-demographic variables and HIV testing. The model included data for the five years under review (2011–2015), and data were adjusted for gender, age, race, education, income, marital status, and healthcare coverage. Some of the variables were merged and then compared with the reference category. For example, the variable marital status was re-categorized into single and couple; those that were divorced, widowed, separated and never married were grouped as single, and compared to those that were in a couple relationship (married, and a member of an unmarried couple). Similarly for age, the categories were collapsed into three (18–34—young adults; 35–54—middle age; and ≥55—older adults). The same was done for race, education, and income. Odds ratios and related 95% confidence intervals were derived from regression analysis. Pair-wise rate differences were examined using bivariate survey-weighted logistic regression. Chi-square test and Monte Carlo Permutation method were used to obtain *p* values. The significance level was set at *p* < 0.05, and all tests were two-sided. Unweighted counts, weighted percentages, and logistic regression analyses were performed using the IBM SPSS Complex Samples version 24 (IBM Corp., Armonk, NY, USA) [25].

### 2.5. Ethical Considerations

BRFSS datasets are publicly accessible and do not contain personally identifiable information. CDC ensures that the process of data collection and release are governed by appropriate rules, regulations, and legislative authorizations [26].

## 3. Results

### 3.1. Socio-Demographic Characteristics and HIV Risk Situations of Respondents

In the BRFSS database, between 2011 and 2015, 31,094 adults in GA responded to the question “Have you ever been tested for HIV?” The respondents were ≥18 years old, predominantly female (63.0%, *n* = 19,545), white (66.7%, *n* = 20,743), college graduates (34.9%, *n* = 10,837), married (51.4%, *n* = 15,988), with an annual income of ≥$50,000 (35.5%, *n* = 11,032), and with some form of healthcare coverage (86.0%, *n* = 26,731) (Table 1). In addition, for the years data were available (2011 and 2012), 97.6% (*n* = 14,142) of respondents did not engage in HIV high-risk behaviors. For all the years under review, the results of the descriptive analyses of socio-demographic categories and having been tested for HIV were statistically significant (*p* < 0.001).

### 3.2. Trends in HIV Testing among Adults in GA, 2011–2015

In GA, there were 11,286 (44.2%) respondents who had ever been tested for HIV. The weighted population estimates of those, by year of interview, and the APC for each variable are shown in Table 2. There was a slight decrease in the percentages of respondents who had ever been tested for HIV, from 45.6% in 2011 to 43.7% in 2015 (APC = −0.98, not significant). There was a significant decrease over time in the number of HIV testers among annual income earners of <$15,000 (APC = −2.29). The percentages of HIV testers over time were stable and APCs were not significant among the other categories of socio-demographic variables.

**Figure 1:**
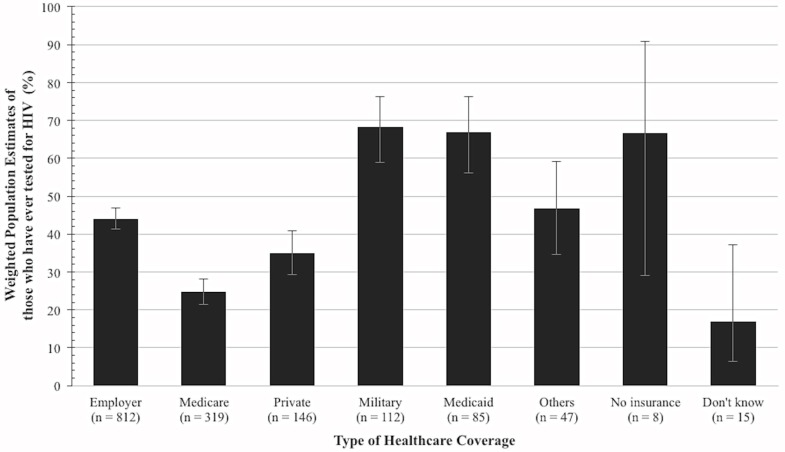
Weighted percentages of adults who have ever tested for HIV in Georgia based on the type of healthcare coverage: BRFSS 2014 data. Note: Error bars indicate 95% confidence intervals for each estimate.

Overall, for the period of 2011–2015 (results not shown in Table 2), approximately 60% of persons between the ages of 25 and 44 had been tested for HIV, compared to <50% of those aged 18–24 and greater than 45 years of age (*p* < 0.001). Also, the highest percentages of testers were among NH black respondents (62.4% vs. 35.2% white and 42.8% Hispanic (*p* < 0.001). Fewer persons with healthcare coverage compared with those with no coverage (41.9% and 52.8%, respectively, *p* < 0.001); and almost 50% of respondents with greater than high school education, earning annual income of <$15,000 (50%), separated (67%), or in an unmarried couple relationship (52%) had been tested for HIV. More persons engaged in high-risk behaviors had been tested for HIV than those who had not (71.8% vs. 43.7%; *p* < 0.001). Data for HIV high-risk activities and for the type of healthcare coverage were available for only 2011–2012 and 2014, respectively. As shown in Figure 1, respondents on the military (Tricare) plan were the highest testers (68.2% (95% CI = 58.9%–76.2%)), followed by those on Medicaid (66.9% (95% CI = 56.1%–76.1%)).

### 3.3. Socio-Demographic Determinants of HIV Testing in GA

In Table 3 are the results of logistic regression analyses for the association between socio-demographic factors and the dependent variable, having ever been tested for HIV, after adjusting for all the variables in the model. Excluded from the model is the variable HIV high-risk situations, because data were available only for 2011 and 2012. Except for healthcare coverage, all the variables entered had a significant effect on the model. Females (OR = 1.13 (95% CI = 1.04, 1.23); *p* = 0.004), NH black/African American respondents (OR = 2.82 (95% CI = 2.54, 3.12); *p* < 0.001), and respondents of other ethnic groups combined (OR = 0.97 (95% CI = 0.85, 1.12); *p* = 0.70) were more likely to have been tested than males, and NH white respondents, respectively. People who were younger than 55 (18–54 years) were more likely than older people to have tested for HIV (OR = 2.58 (95% CI = 2.31, 2.89); *p* < 0.001). The likelihood of being tested for HIV was also associated with levels of education greater than high school (OR = 1.46 (95% CI = 1.31, 1.63); *p* < 0.001), being single (OR = 1.22 (95% CI = 1.11, 1.34); *p* < 0.001), and earning annual income of less than $50,000 (OR = 1.18 (95% CI = 1.04, 1.34); *p* = 0.01 for <$25,000 and OR = 1.14 (95% CI = 1.01, 1.28); *p* = 0.03 for $25,000–$50,000 annual income), compared to less than high school education, being in a couple relationship, and earning more than $50,000 annually, respectively. Having healthcare coverage was not significantly associated with HIV testing.

## 4. Discussion

BRFSS data from 2011 to 2015 (the years of the most current data) were analyzed to examine the temporal trends and socio-demographic factors associated with HIV testing among adults in GA. Overall in GA, there was a slight decrease in the percentages of respondents who had ever been tested for HIV, from 45.6% in 2011 to 43.7% in 2015, with a non-significant APC. During this time, the annual percentages of those tested were higher for GA compared to the national rates (35.9% in 2011, 38.0% in 2015). The factors associated with HIV testing included being female, black/African American, single, younger than 55 years, having greater than high school education, and earning $50,000 or less annually.

The results of the current study show that the HIV testing trends were stable between 2011 and 2015, however, less than half of the adults living in GA had been tested for HIV. Results of earlier studies conducted nationally and in other parts of the U.S. [27,28] show that the percentages of adults who had ever tested for HIV increased significantly between 2000 and 2010 (36.6% in 2000, 45.0% in 2010, *p* < 0.0001) [27]. A study that analyzed data from the Southeastern Pennsylvania Household Health Survey between 2002 and 2010 to evaluate HIV testing over time, reported that testing trends increased among all demographic groups, but existing differences in testing before 2006 persisted after that year as follows: younger patients, racial/ethnic minorities, and patients on Medicaid were all more likely to get tested than their counterparts [28].

Barriers to HIV testing include HIV-related stigma, sexuality, religion, race, and class, emphasizing responsibility, testing concerns, and media influences [29,30]. The percentages of respondents who had been tested for HIV were highly associated with the presence of HIV risk factors and with self-reported current risks of contracting HIV. Racial minorities, younger persons, especially young black/African American (men having sex with men (MSM)), have the highest risk and prevalence of HIV/AIDS [28]. Gay, bisexual, and other men who have sex with men accounted for an estimated 2% of the total population, and 55.0% of people living with HIV in the United States in 2013 [31]. Georgia is among the states with the highest population of MSM and African Americans [7,32], and this may account for the higher rate of HIV testers in GA, compared to the national rate. HIV risk is associated with low socio-economic status among heterosexual populations [33] and the current study shows that lower income earners and those without healthcare coverage were more likely to test for HIV. However, this does not explain why the participants that attained educational levels greater than high school tested more than those with high school or lesser education. A possible explanation may be that a great proportion of the study participants were educated beyond high school.

A similar study by Handel et al. (2016), analyzed the BRFSS data for 2011–2013, and reported that a national average of 33% of young adults (18–24 years) had tested for HIV [34]; the average for the same age group in GA was approximately 40.0% as reported by the current study. The Handel study also showed that a significant decrease in the prevalence of HIV testing was detected overall from 42.4% in 2011 to 39.5% in 2013 among young adult females nationally, with significant racial/ethnic differences in the rates of decline (9.0% decrease among young adult black females, and 3.3% decrease among young adult white females).

A disadvantage of low HIV testing among persons who are perceived as low-risk is the missing of opportunities to diagnose HIV-infected persons and linking them to care. Reasons for fewer people with healthcare coverage not being tested may be because (a) routine HIV testing is not offered in the places where most people get their health care and (b) awareness of CDC’s 2006 recommendations for HIV screening has been low among primary care providers [35,36]. Release of the United States Preventive Services Task Force recommendations for HIV testing in 2013, and the provision of the Affordable Care Act that both HIV screening and targeted risk-based testing are now covered without cost-sharing as part of the essential benefits package, may boost future HIV testing rates [36].

A strength of the present report is the utilization of the most currently available BRFSS data. It is also among the few studies that have examined the trends of HIV testing with the APCs, and the associated socio-demographic factors of HIV testing and is the only report solely for the state of GA. There are some limitations. The BRFSS data are self-reported by respondents and are subject to recall bias. The survey is based on non-institutionalized populations and excludes persons with the same risk of exposure who are residing elsewhere, such as nursing homes or long-term-care facilities. Since data are collected by telephone, individuals who live in households without a residential telephone or cell phone are not included. Further, the sampling frame of the BRFSS is the entire state; therefore, some rural areas might be represented by relatively few interviews. Because of these limitations, the results might be either underestimated or overestimated. Despite these limitations, data from the BRFSS are reliable and generally valid because the content of the survey questions, questionnaire design, data collection, procedures, interviewing techniques, and data processing have been developed to improve data quality [37].

## 5. Conclusions

Between 2011 and 2015, the percentage of adults in GA who have ever been tested for HIV has remained stable, with less than 50% now reporting to have been tested. In GA, increasing access to and utilization of HIV testing should be a public health priority, and more programs that will increase awareness to recommendations for testing among healthcare providers are warranted.

## Figures and Tables

**Table 1:** Socio-demographic characteristics and HIV risk situations of survey respondents to the question “Have you ever been tested for HIV?” in Georgia: 2011–2015 BRFSS data.

	Total (*N* = 31,094)	2011 (*n* = 8977)		2012 (*n* = 5554)		2013 (*n* = 7010)		2014 (*n* = 5551)		2015 (*n* = 4002)		*p*-Value
	N	n	%	n	%	n	%	n	%	n	%	
Gender												<0.001
Male	11,549	3162	35.2	2062	37.1	2621	37.4	2132	38.4	1572	37.1	
Female	19,545	5815	64.8	3492	62.9	4389	62.6	3419	61.6	2430	62.9	
Age (years)												<0.001
18–24	1495	309	3.4	283	5.1	428	6.1	290	5.2	185	4.6	
25–34	2985	828	9.2	540	9.7	775	11.1	485	8.7	357	8.9	
35–44	3914	1170	13.0	697	12.6	943	13.4	658	11.9	446	11.2	
45–54	5605	1683	18.8	1037	18.7	1281	18.3	950	17.1	654	16.4	
55–64	7248	2177	24.3	1263	22.7	1630	23.2	1260	22.7	918	22.9	
65+	9847	2810	31.3	1734	31.2	1953	27.9	1908	34.4	1442	36.0	
Race												<0.001
White, NH	20,743	6192	69.0	3687	66.4	4504	64.2	3678	66.2	2682	69.0	
Black, NH	7413	2012	22.4	1340	24.1	1766	25.2	1349	24.3	946	22.4	
Hispanic	1212	341	3.8	206	3.7	308	4.4	217	3.9	140	3.8	
Other, NH	850	216	2.4	187	3.4	210	3.0	120	2.2	117	2.4	
Multiracial, NH	445	106	1.2	74	1.3	117	1.7	94	1.7	54	1.2	
Do not know/Refused	431	110	1.2	60	1.1	105	1.5	93	1.7	63	1.2	
Education												<0.001
<High school	3497	997	11.1	694	12.4	771	11.0	615	11.1	420	10.5	
High School/GED	8593	2550	28.4	1605	28.9	1923	27.4	1469	26.5	1046	26.1	
Some Post High School	8086	2314	25.8	1442	26.0	1825	26.1	1474	26.5	1031	25.8	
College Grad	10,837	3090	34.4	1797	32.4	2477	35.3	1980	35.7	1493	37.3	
Do not know/Refused	81	26	0.3	16	0.3	14	0.2	13	0.2	12	0.3	
Annual Income (USD ($))												<0.001
<15,000	3511	995	11.1	697	12.6	825	11.8	610	11.0	384	9.6	
15,000–<25,000	5215	1536	17.1	996	17.9	1122	16.0	930	16.7	631	15.8	
25,000–<35,000	3260	998	11.1	595	10.7	738	10.5	564	10.2	365	9.1	
35,000–<50,000	3650	1079	12.0	625	11.3	856	12.2	640	11.5	450	11.2	
50,000+	11,032	3142	35.0	1917	34.5	2451	35.0	2043	36.8	1479	37.0	
Do not know/Refused	4426	1227	13.7	724	13.0	1018	14.5	764	13.8	693	17.3	
Marital status												<0.001
Married	15,988	4835	53.9	2845	51.2	3461	49.4	2787	50.2	2060	51.5	
Divorced	4571	1313	14.6	798	14.4	1073	15.3	808	14.5	579	14.5	
Widowed	4482	1312	14.6	830	14.9	903	12.9	858	15.5	579	14.5	
Separated	838	226	2.5	138	2.5	223	3.2	151	2.7	100	2.5	
Never married	4430	1090	12.1	824	14.8	1142	16.3	809	14.6	565	14.1	
A member of an unmarried couple	648	170	1.9	103	1.9	171	2.4	111	2.0	93	2.3	
Refused	137	31	0.4	16	0.3	37	0.5	27	0.5	26	0.6	
Healthcare coverage												<0.001
Yes	26,731	7680	85.6	4695	84.5	5872	83.8	4866	87.6	3618	90.4	
No	4269	1272	14.2	847	15.3	1116	15.9	664	12.0	370	9.2	
Do not know/Refused	94	25	0.2	12	0.2	22	0.3	21	0.4	14	0.4	
HIV high risk situations	*N* = 14,497	*n* = 8959		*n* = 5538								<0.001
Yes	309	185	2.1	124	2.2	N/A	N/A	N/A	N/A			
No	14,142	8748	97.6	5394	97.4	N/A	N/A	N/A	N/A			
Do not know/Refused	46	26	0.3	20	0.4	N/A	N/A	N/A	N/A			

Acronyms: NH—non-Hispanic, GED—General Educational Development, N/A—data not available, USD—United States Dollar.

**Table 2:** Weighted percentages and annual percent change of adults who have ever been tested for HIV in Georgia by year of interview: 2011–2015 BRFSS data.

	Total	2011		2012		2013		2014		2015		
	Unwt. N	Unwt. N	Wt. % (95% CI)	Unwt. N	Wt. % (95% CI)	Unwt. N	Wt. % (95% CI)	Unwt. N	Wt. % (95% CI)	Unwt. N	Wt. % (95% CI)	APC *
Overall GA	11,286	3174	45.6 (44.0, 47.2)	1961	44.3 (42.4, 46.1)	2753	43.6 (42.0, 45.3)	1990	43.7 (41.9, 45.5)	1408	43.7 (41.6, 45.9)	−0.98
Gender												
Male	4376	1145	45.0 (42.5, 47.6)	746	43.0 (40.1, 45.9)	1101	43.6 (41.1, 46.2)	802	41.9 (39.2, 44.7)	582	43.3 (40.0, 46.5)	−1.02
Female	6910	2029	46.1 (44.1, 48.0)	1215	45.5 (43.1, 47.8)	1652	43.6 (41.6, 45.6)	1188	45.4 (43.1, 47.7)	826	44.2 (41.3, 47.0)	−0.86
Age (years)												
18–24	666	140	42.4 (36.3, 48.8)	352	46.2 (39.9, 52.7)	191	39.5(34.3, 45.0)	122	40.4 (34.5, 46.6)	73	40.1 (32.6, 48.1)	−2.43
25–34	1902	540	65.4 (61.1, 69.6)	352	62.5 (57.2, 67.4)	493	62.6 (58.2, 66.9)	297	60.9 (55.8, 65.8)	220	62.9 (56.9, 68.6)	−1.03
35–44	2401	712	62.0 (58.4, 65.4)	397	57.2 (52.6, 61.7)	594	60.7 (56.7, 64.6)	411	60.1 (55.5, 64.6)	287	61.8 (56.3, 67.1)	0.43
45–54	2656	769	46.0 (43.0, 49.0)	494	49.3 (45.4, 53.2)	615	44.4 (40.9, 47.9)	461	46.8 (42.9, 50.7)	317	47.9 (43.3, 52.5)	0.29
55–64	2264	653	31.9 (29.4, 34.5)	352	28.8 (25.6, 32.3)	540	33.5 (30.6, 36.6)	406	33.9 (30.6, 37.4)	313	32.6 (29.0, 36.4)	2.09
65+	1397	360	13.9 (12.3, 15.7)	226	14.6 (12.4, 17.1)	320	16.0 (14.0, 18.2)	293	15.8 (13.9, 18.0)	198	15.6 (13.2, 18.4)	3.15
Race												
White, NH	6058	1716	36.9 (35.1, 38.8)	983	33.4 (31.2, 35.6)	1460	36.3 (34.5, 38.2)	978	34.3 (32.2, 36.5)	753	35.2 (32.8, 37.7)	−0.68
Black, NH	4201	1127	65.3 (62.1, 68.4)	753	65.6 (62.1, 68.9)	970	59.3 (56.0, 62.6)	772	63.4 (59.9, 66.8)	490	58.3 (53.9, 62.8)	−2.58
Hispanic	549	155	40.8 (33.8, 48.1)	88	42.0 (34.0, 50.5)	138	43.9 (37.5, 50.5)	95	38.1 (30.8, 46.0)	67	49.7 (39.5, 60.0)	3.02
Multiracial, NH	237	52	54.2 (41.4, 66.5)	43	59.5 (44.9, 72.7)	60	55.0 (42.2, 67.3)	53	57.1 (42.7, 70.4)	33	69.9 (54.3, 81.6)	4.79
Other, NH	326	79	39.1 (30.6, 48.3)	75	40.2 (31.4, 49.6)	82	29.8 (20.3, 39.3)	49	39.7 (28.4, 51.0)	41	37.1(26.9, 48.5)	−1.17
Do not know/Refused	172	45	53.6 (39.8, 66.8)	19	39.3 (22.5, 59.0)	43	45.5 (34.0, 57.6)	43	53.6 (39.9, 66.8)	24	43.2 (26.8, 61.3)	-
Education												
<High School	1051	280	40.2 (35.5, 45.1)	207	38.0 (32.8, 43.4)	235	36.2 (31.5, 41.3)	181	34.6 (29.4, 40.1)	127	42.0 (35.2, 49.0)	−0.06
HS/GED	2722	788	42.0 (39.0, 45.0)	493	40.6 (37.1, 44.1)	628	39.5 (36.5, 42.6)	440	40.1 (36.8, 43.6)	324	40.4 (36.3, 44.5)	−0.90
Some Post HS	3371	913	51.8 (48.8, 54.7)	559	47.8 (44.4, 51.3)	820	48.9 (45.9, 52.0)	602	50.3 (47.0, 53.6)	397	46.3 (42.2, 50.4)	−1.72
College Grad	4379	1185	46.5 (44.0, 48.9)	696	48.3 (45.3, 51.5)	1066	47.0 (44.5, 49.5)	766	46.0 (43.2, 48.8)	558	45.8 (42.7, 49.1)	−0.79
Do not know/Refused	20	8	44.9 (17.4, 75.8)	6	58.7 (29.5, 82.8)	4	40.2 (14.6, 72.6)	1	3.1 (0.4, 21.7)	2	8.8 (1.4, 39.2)	-
Annual income (USD ($))												
<15,000	1476	394	52.6 (47.5, 57.6)	308	51.1 (45.6, 56.6)	353	49.9 (45.3, 54.5)	249	47.3 (41.4, 53.2)	150	48.7 (41.4, 56.0)	−2.29 *
15,000–<25,000	1995	570	49.7 (45.7–53.6)	350	46.8 (42.3–51.4)	475	46.7 (42.7–50.7)	340	42.6 (38.2, 47.1)	231	46.7 (41.2, 52.3)	−2.16
25,000–<35,000	1171	335	45.3 (40.5–50.2)	201	45.0 (39.2–50.9)	267	46.6 (41.5–51.8)	201	46.3 (40.7, 52.0)	111	40.4 (33.7, 47.5)	−1.98
35,000–<50,000	1332	377	46.1 (41.6, 50.6)	206	42.3 (37.1, 47.7)	337	47.4 (42.7, 52.1)	232	47.2 (42.0, 52.5)	160	49.4 (43.1, 55.8)	2.51
50,000+	4461	1197	45.0 (42.5, 47.4)	710	43.5 (40.5, 46.5)	1034	42.5 (40.0, 45.1)	771	43.7 (40.9, 46.5)	562	43.9 (40.6, 47.2)	−0.45
Do not know/Refused	1108	301	34.9 (30.3, 39.8)	186	36.1 (30.9, 41.7)	287	32.1 (27.8, 36.7)	197	36.8 (31.8, 42.1)	194	35.1 (29.8, 40.7)	-
Marital status												
Married	5534	1586	41.1 (39.2, 43.1)	890	36.6 (34.3, 39.0)	1218	38.4 (36.3, 40.5)	919	39.1 (36.8, 41.5)	654	37.6 (34.9, 40.4)	−1.11
Divorced	2116	574	51.6 (47.6, 55.6)	346	55.0 (50.1, 59.8)	540	56.4 (51.9, 60.8)	363	53.1 (48.2, 57.8)	257	54.2 (48.6, 59.7)	0.63
Widowed	704	204	21.6 (18.3, 25.2)	127	21.7 (17.4, 26.7)	166	20.6 (17.2, 24.6)	151	22.8 (18.4, 27.9)	98	29.7 (23.8, 36.4)	7.10
Separated	512	143	68.5 (59.4, 76.4)	87	69.5 (58.7, 78.6)	140	68.9 (60.0, 76.6)	88	66.5 (55.4, 76.0)	56	62.5 (49.1, 74.1)	−2.25
Never married	2255	553	53.7 (49.5, 57.9)	440	54.8 (50.3, 59.1)	585	50.2 (46.3, 54.1)	400	50.4 (46.0, 54.8)	290	53.3 (47.9, 58.5)	−0.98
A member of an unmarried couple	366	100	53.6 (43.9, 63.1)	64	61.5 (48.9, 72.6)	90	50.3 (41.4, 59.1)	59	50.2 (38.8, 61.5)	43	44.2 (33.0, 55.9)	−5.72
Refused	56	14	39.5 (18.4, 65.4)	7	35.6 (12.0, 69.1)	14	42.5 (23.5, 64.0)	10	53.4 (28.6, 76.7)	10	29.4 (13.3, 53.1)	-
Health care coverage												
Yes	9216	2541	43.0 (41.3, 44.7)	1524	41.2 (39.3, 43.2)	2157	40.6 (38.9, 42.3)	1662	42.2 (40.4, 44.1)	1215	42.4 (40.1, 44.6)	−0.04
No	2303	629	53.6 (49.6, 57.6)	435	54.8 (50.2, 59.3)	584	53.9 (49.9, 57.8)	324	49.5 (44.6, 54.4)	187	51.2 (44.6, 57.7)	−1.91
Do not know/Refused	24	4	12.6 (2.3, 43.3)	2	23.4 (2.6, 77.4)	12	51.0 (20.4, 82.2)	4	37.0 (9.6, 76.4)	6	43.9 (8.0, 71.6)	-
HIV high risk situations	*N* = 5114	*n* = 3164		*n* = 1950								
Yes	211	130	74.0 (63.8, 82.1)	81	69.4 (58.1, 78.7)	N/A	N/A	N/A	N/A	N/A	N/A	-
No	4884	3020	44.2 (42.6, 45.8)	1864	43.2 (41.3, 45.1)	N/A	N/A	N/A	N/A	N/A	N/A	-
Do not know/Refused	19	14	75.3 (41.8, 90.0)	5	25.0 (6.0, 49.4)	*N/A*	*N/A*	*N/A*	*N/A*	N/A	N/A	-
Overall USA	608,484	132,471	35.9 (35.6, 36.2)	128,927	35.3 (35.0, 35.5)	130,922	35.9 (35.6, 36.2)	115,866	34.4 (34.1, 34.7)	113,779	38.0 (37.7, 38.3)	0.88

Acronyms: Unwt. N—unweighted counts, Wt. %—weighted population estimates, CI—confidence interval, APC—Annual Percent Change, NH—non-Hispanic, GED—General Educational Development, N/A—data not available. Note: *p*-values were significant for the association between all the socio-demographic categories and having ever been tested for HIV. * APC was not significantly different from zero at alpha = 0.05 for all the variables, except annual income <$15,000. (-) APC was not calculated for respondents who do not know or refused to respond to questions asked, and for HIV high risk situations. USD—United States Dollar.

**Table 3:** Logistic regression analysis of socio-demographic factors associated with having ever been tested for HIV in Georgia: BRFSS 2011–2015.

Variable	Odds Ratio	95% CI	*p*-Value
Gender			
Female	1.134	1.041, 1.234	0.004
Male	Reference		
Age (years)			
18–34	2.583	2.306, 2.894	<0.001
35–54	2.578	2.344, 2.836	<0.001
≥55	Reference		
Race			
Black, NH	2.815	2.538, 3.122	<0.001
Others	0.973	0.845, 1.120	0.701
White, NH	Reference		
Education			
College graduate	1.463	1.313, 1.631	<0.001
Some post high school	1.462	1.311, 1.630	<0.001
≤HS	Reference		
Income			
<25,000	1.178	1.035, 1.342	0.013
25,000–50,000	1.138	1.014, 1.277	0.028
>50,000	Reference		
Marital status			
Single	1.216	1.106, 1.337	<0.001
Couple	Reference		
Healthcare coverage			
No	1.046	0.927, 1.179	0.467
Yes	Reference		
